# From the Vineyard to the Winery: How Microbial Ecology Drives Regional Distinctiveness of Wine

**DOI:** 10.3389/fmicb.2019.02679

**Published:** 2019-11-20

**Authors:** Di Liu, Pangzhen Zhang, Deli Chen, Kate Howell

**Affiliations:** School of Agriculture and Food, Faculty of Veterinary and Agricultural Sciences, The University of Melbourne, Melbourne, VIC, Australia

**Keywords:** wine quality, microbial biogeography, climate, soil, bacteria, fungi

## Abstract

Wine production is a complex process from the vineyard to the winery. On this journey, microbes play a decisive role. From the environment where the vines grow, encompassing soil, topography, weather and climate through to management practices in vineyards, the microbes present can potentially change the composition of wine. Introduction of grapes into the winery and the start of winemaking processes modify microbial communities further. Recent advances in next-generation sequencing (NGS) technology have progressed our understanding of microbial communities associated with grapes and fermentations. We now have a finer appreciation of microbial diversity across wine producing regions to begin to understand how diversity can contribute to wine quality and style characteristics. In this review, we highlight literature surrounding wine-related microorganisms and how these affect factors interact with and shape microbial communities and contribute to wine quality. By discussing the geography, climate and soil of environments and viticulture and winemaking practices, we claim microbial biogeography as a new perspective to impact wine quality and regionality. Depending on geospatial scales, habitats, and taxa, the microbial community respond to local conditions. We discuss the effect of a changing climate on local conditions and how this may alter microbial diversity and thus wine style. With increasing understanding of microbial diversity and their effects on wine fermentation, wine production can be optimised with enhancing the expression of regional characteristics by understanding and managing the microbes present.

## Introduction

Wine production is a global billion-dollar industry for which regionally distinct wine characteristics, collectively known as “*terroir*,” are an important collection of traits that wine industry would like to define. Wines made from the same grape cultivar but grown in different regions are appreciated for their distinctive features (Van Leeuwen and Seguin, 2006). Regionality can be identified with chemical compositions and sensory properties (Pereira et al., 2005; Gonzálvez et al., 2009; Robinson et al., 2012), and are likely to be ascribed to local environmental parameters that influence grapevine growth and development, such as soil types, climate, topography and human management, but the mechanisms by which these factors affect wine composition remain tenuous (Van Leeuwen and Seguin, 2006; Gladstones, 2011; Vaudour et al., 2015).

**FIGURE 1:**
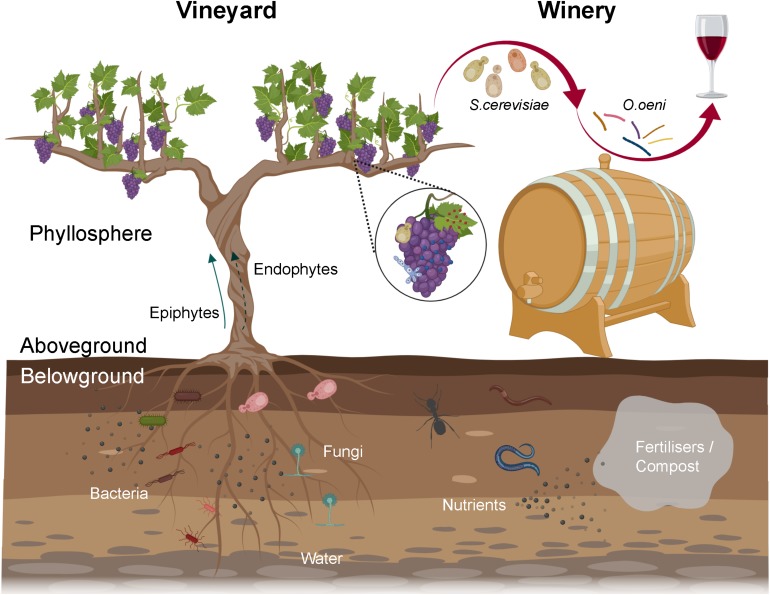
Overview of the wine-related microbiota from the vineyard to the winery. Microbiota associated with grapevine phyllosphere, especially grapes, can enter musts and constitute wine microbial consortium, in which fermentative yeasts and LAB conduct alcoholic and malolactic fermentation, respectively. The rhizosphere harbours diverse microbes that can benefit plants by enabling nutrient uptake and tolerance to (a)biotic stress. Soil borne microorganisms might translocate to the phyllosphere internally (endophytes) or externally (epiphytes), thereby entering wine fermentation. Viticulture practices, for example fertilisers/compost addition, can modify soil microbiota via shifting nutrient pools or adding manure borne microorganisms (created with BioRender, https://app.biorender.com/).

**FIGURE 2:**
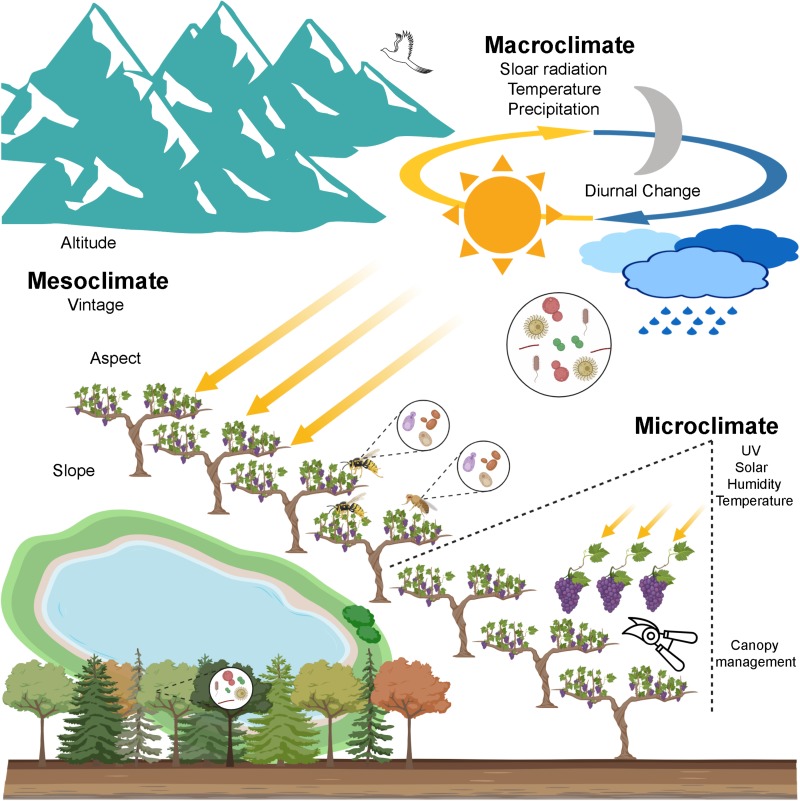
A scenario of wine microbial biogeography. Grapevine-associated microbiota originates from the local ecosystem encompassing soil, air, precipitation, native forests, etc. Genetic isolation is one driver of the geographic pattern that long distance decreases the gene flow that depends on physical forces and animal vectors (e.g., insects and birds). Climate is a profound environmental element shaping the microbial geographic pattern and thus affects wine quality. Macroclimate exerts influences on the regional pattern of bacteria and fungi. Mesoclimate at the vineyard scale shows weaker influences on the microbial distribution, especially for bacteria. Microclimate within the grapevine, modified by canopy management, may influence associated microbiota, this still remains to be shown (created with BioRender, https://app.biorender.com/).

From the vineyard to the winery, microorganisms play key roles in wine production and quality. The grapevine (*Vitis vinifera*) phyllosphere harbours diverse microbes including yeasts, filamentous fungi and bacteria that substantially modulate grapevine health, growth, and grape and wine production (Figure 1; Barata et al., 2012; Gilbert et al., 2014). Microbes could originate from the vineyard soil (Zarraonaindia et al., 2015; Morrison-Whittle and Goddard, 2018), air, precipitation (rainfall, hail, snow), be transported by animal vectors (bees, insects, and birds) (Goddard et al., 2010; Francesca et al., 2012; Stefanini et al., 2012; Lam and Howell, 2015), and be resident in nearby native forests (Figures 1, 2; Knight and Goddard, 2015; Morrison-Whittle and Goddard, 2018). Microbes that are grapevine-associated and are transferred to the must have a profound influence on wine composition, flavour and quality (Figure 1; Barata et al., 2012). Fermentative yeasts (primarily *Saccharomyces cerevisiae*) and lactic acid bacteria (LAB, predominantly *Oenococcus oeni*) in the must modulate the flavour and aroma of wine (Swiegers et al., 2005). Beyond these species, many microorganisms in the must could release metabolites changing the chemical environment and/or fermentation processes and thus affect wine compositions and characteristics, for example, release of inhibitory molecules altering *Saccharomyces* metabolism (Swiegers et al., 2005; Bokulich et al., 2016).

Increasing evidence supports a microbial aspect to wine regionality may be due, in part, to regionally structured microbial communities, or microbial biogeography. Advanced genetic-based methodologies, in particular next-generation sequencing (NGS), have allowed researchers to sample microbial diversity more deeply and widely and encouraged more comprehensive biogeographical surveys. This has marked the beginning of a new era of information on the grapevine-associated microbiome across multiple scales (region, vineyard, and vine) to elucidate wine quality and regional variation (Bokulich et al., 2014; Taylor et al., 2014; Burns et al., 2015; Morrison-Whittle and Goddard, 2018). This review aims to disentangle the role of microbial biogeography on wine production, by considering how microbes interact with environmental conditions and thus drive wine quality and style.

We commence our discussion with a review of current knowledge on microbial geographic patterns and how they differ from the vineyard to the winery, and then describe how climate, soil, and anthropogenic practices can affect microbial communities through the winemaking process to the finished wine. The concept of scale is crucial to define microbial biogeography, as large-scale geographic and climatic features significantly affect microbial communities, but at smaller scales these differences may not be apparent. We propose that microbial biogeography gives a theoretical basis to wine *terroir*, which further provides information to the industry to produce distinctive and quality wines through microbial manipulation. This is particularly important as wine styles are altered by weather patterns and we conclude by considering how microbial biogeography and activity may respond to changing climates.

## Microbial Biogeography: A Microbial Insight to Understand Variation in Wine Quality

Global studies of microbial biogeography have shown that distinct microbial populations are present in soil, bodies of water and ocean biomes and associated with plants (Martiny et al., 2006). Soil fungi and bacteria show global niche differentiation that is associated with contrasting diversity responses to environmental filtering (for example by precipitation, soil pH) and biotic interactions (Bahram et al., 2018). Culture-based microbiological methods revealed only a small portion of the diversity of environments (Martiny et al., 2006), and so previous studies on the vineyard and wine microbiome do not reveal regional boundary restrained patterns (Barata et al., 2012). New technologies such as NGS benefit from being culture-independent and can therefore reveal a distinct scenario of microbial biogeography. We propose that geographic patterns favour fungi- or bacteria-driven metabolites and thus contribute to wine composition and quality. Some key wine microbial biogeography studies are listed in Table 1, where we highlight the influence of climate, soil and anthropogenic practices (a comparative list of these studies is given in Supplementary Table S1).

**TABLE 1:** Recent findings on wine microbial ecology from the vineyard to the winery.

**Microorganisms**	**Scale**	**Habitat**	**Methodology**	**Major Conclusions**	**References**
Yeasts	Three regions	Grape juice	Culture-dependent method, ITS-RFLP and D1/D2 26S sequencing	(i) Regional delineations were found on yeast communities and *S. cerevisiae* populations	Gayevskiy and Goddard, 2012
				(ii) Reasonable levels of gene flow were found in *S. cerevisiae* populations among regions	
Bacteria, fungi	Four regions	Grape must	Culture-independent method, 16S rRNA and ITS amplicon sequencing	(i) Regional origin defined grape must microbial patterns, with some influences by the cultivar	Bokulich et al., 2014
				(ii) Weather and climate were responsible for driving biogeographical diversity	
				(iii) Vintage exerted seasonal shifts in grape microbiota within single vineyards, especially bacteria	
*S. cerevisiae*	Six regions	Vineyard soil, grape juice, native forest soil and fruits	Culture-dependent method, microsatellite loci amplification and genotyping	(i) Regionally genetically differentiated *S. cerevisiae* populations drove different wine phenotype	Knight and Goddard, 2015; Knight et al., 2015
				(ii) Genetic similarity of *S. cerevisiae* populations was found between vineyards and forests within regions	
Fungi	Within region, three vineyards	Grapes	Culture-dependent method, ITS-ARISA fingerprinting	(i) Intravineyard variations were greater than intervineyard variations, possibly due to microclimate’s influences on grape microbiota	Setati et al., 2012
				(ii) The least treated vineyard (biodynamic and integrated) displayed significantly higher fungal species richness	
Bacteria	Within region, seven vineyards	Grape must, end malolactic ferments	Culture-independent method, 16S rRNA amplicon sequencing	(i) Bacterial community heterogeneities were influenced by the cultivar and geographic orientation	Portillo et al., 2016
				(ii) Intervineyard variations were greater than intravineyard variations	
Bacteria	Within region, 19 vineyards	Soil	Culture-independent method, 16S rRNA amplicon sequencing	(i) Soil bacterial communities were structured with respect to soil properties, location, geographic features, and management practices, e.g., conventional/organic/biodynamic systems	Burns et al., 2015; Burns et al., 2016
				(ii) High relative abundances of the majority of dominant taxa were found in soils with lower carbon or nitrogen contents	
Bacteria	Within region, five vineyards	Soil, roots, leaves, flowers/grapes	Culture-independent method, 16S rRNA amplicon sequencing	(i) Most grapevine OTUs originated in the soil	Zarraonaindia et al., 2015
				(ii) Soil-borne bacteria were selected by plants	
				(iii) Microbial structure was influenced by edaphic factors, i.e., pH, C:N ration, soil carbon, etc.	
Fungi	Six regions	Vineyard soil, bark, juice and ferments, native forest soil and fruits	Culture-independent method, 26S rRNA amplicon sequencing	(i) Vineyard fungi accounted for ∼40% of the diversity in juice and ferments	Morrison-Whittle and Goddard, 2018
				(ii) The geographical diversification of must microbiome weakened during fermentation	
Fungi	Within region, 12 vineyards	Soil, bark, grapes, juice and ferments	Culture-independent method, 26S rRNA amplicon sequencing	Biodynamic practices significantly affected soil and grapevine-associated microbiome but not the harvest juice communities, nor on final wine quality	Morrison-Whittle and Goddard, 2017
Fungi	Within vineyard	Grapes, must and ferments	Culture-independent method for fungi with 18S rRNA amplicon sequencing, culture-dependent method for yeasts	(i) Lower biodiversity of yeasts and fungal populations was measured in organically- than conventionally-farmed grapes and ferments	Grangeteau et al., 2017
				(ii) SO_2_ addition favoured the domination of *S. cerevisiae* during fermentation	
Yeasts	Within vineyard	Must and ferments	Culture-dependent method, D1/D2 26S sequencing	Prefermentative cold soak modified yeast dynamics in a temperature-dependent manner	Maturano et al., 2015
Bacteria, fungi	Within vineyard	Must and ferments	Culture-independent method, 16S rRNA and ITS amplicon sequencing	SO_2_ treatment altered wine microbial diversity in a dose-dependent manner	Bokulich et al., 2015

### Microbiota-Metabolome Geographic Patterns to Elucidate Wine Regionality

Geographic delineations of *S. cerevisiae* populations and cultivable yeasts were first reported in New Zealand vineyards, providing evidence for regional distribution of yeast populations (Table 1; Gayevskiy and Goddard, 2012). In the United States, Bokulich et al. (2014) used NGS of 16S rRNA and internal transcribed spacer (ITS) ribosomal sequence to demonstrate regionally structured bacterial and fungal consortia in grape musts, with some influences from cultivar and vintage (Table 1). These studies posit that microbial biogeography is a contributor to wine regionality expression. Further biogeographical correlations between wine-related microbiota and regional origins have been reported, holding for newly planted or older vineyards and are representative from vineyards planted around the world (Tofalo et al., 2014; Pinto et al., 2015; Wang et al., 2015; Bokulich et al., 2016; Capece et al., 2016; Garofalo et al., 2016; Marzano et al., 2016; Portillo et al., 2016; El Khoury et al., 2017; Mezzasalma et al., 2017, 2018; Singh et al., 2018; Vitulo et al., 2019). Notably, the microbial geographic diversification in the must weakens as fermentation processes, due to *S. cerevisiae* yeasts dominance breaks the community diversity (Morrison-Whittle and Goddard, 2018). In addition, *S. cerevisiae* can persist perennially in a particular vineyard or winery within one single region, thereby enabling wine style consistency between vintages (Börlin et al., 2016; Guzzon et al., 2018).

The geographic diversification observed in microbiota has been verified in wine chemical attributes (Knight et al., 2015; Bokulich et al., 2016). Knight et al. (2015) empirically showed that regionally differentiated *S. cerevisiae* populations drove different metabolites in the resultant wines (Table 1; Knight et al., 2015). Further, microbial patterns correlating with regional metabolite profiles were reported by Bokulich et al. (2016), and showed the importance of the fermentative yeasts (for example, *S. cerevisiae*, *Hanseniaspora*, *Pichia*) and LAB (*Leuconostocaceae*). These correlations between microbiome and metabolome can be used to predict wine compositions and styles with their microbial patterns and deserve future study. For example, exploration of non-*Saccharomyces* yeasts that enhance wine aroma complexity, the importance of microbial diversity in early fermentation, and the likelihood of significant interactions between yeasts and bacteria will held unpick mechanisms to explain the correlations described in the studies above.

### Geospatial Scales Shape Geographic Diversification of Microbes

The biogeographic patterns of microbes in the wine environment are generally based on regional scales which are interpreted differently within each winegrowing country. This means that the notion of a “region” is not strictly defined and varies considerably. For example, a wine region can describe an association of vineyards spanning hundreds or even thousands of kilometres (Gayevskiy and Goddard, 2012; Bokulich et al., 2014; Taylor et al., 2014; Knight and Goddard, 2015) or be quite a small geographic area (Pinto et al., 2015). When comparing microbial communities in smaller geographical scales (at the scale of individual vineyards), the geographical patterns among populations are more evident for fungi than for bacteria (Bokulich et al., 2014; Miura et al., 2017). The leaf and grape fungal community dissimilarities between sampling sites increase as geographic distance increases (Miura et al., 2017).

Non-mobile yeasts require animals (insects and birds) to be transferred across regions (Figure 2; Francesca et al., 2012; Stefanini et al., 2012; Lam and Howell, 2015), animal vectors are one potential biotic factor shaping the yeast and fungal community dissimilarities. When the studied scale is a small area, more factors maybe involved in the uniqueness of a site. Grape varieties and clones exert marked impact on grape surface bacteria within vineyard. For example, *Bacteroidetes*, *Chloroflexi*, *Acidobacteria*, and *Planctomycetes* are clone- specific phyla alongside the prevalent phylum *Cyanobacteria*, *Proteobacteria*, and *Firmicutes* (Zhang et al., 2019). This is in contrast with findings by Bokulich et al. (2014) who showed that the cultivar-specific influence on microbial diversity is weak in larger scales. While Portillo et al. (2016) reported higher variability of bacteria between vineyards compared to intra-vineyard, Setati et al. (2012) demonstrated that greater intra-vineyard variation was evident than inter-vineyard based on fungal populations, including yeasts (Table 1). In these cases, microclimate may play a more important role in structuring fungal communities (details in the section “Microclimate”), but this supposition requires further empirical studies for confirmation.

Microbial biogeography is indelibly shaped by genetic isolation, as gene flow decreases with longer distances and depends on vectors (Martiny et al., 2006; Kuehne et al., 2007). Likewise, wine-related microbiota from relatively small scales are more similar to one another than those from a larger geographic areas (Martínez et al., 2007; Miura et al., 2017), although whether microbial patterns present a distance-dependant model is not clear. At the same time, adaptation to local environments influences how microbial ecology develops and diversifies (Martiny et al., 2006). For example, *S. cerevisiae* shows remarkable ability to adapt and thrive in human-associated food fermentative ecosystems (Legras et al., 2018). *S. cerevisiae* could be dispersed within, and successfully co-exist with other yeasts over a very small scale (10–200 m from the winery) (Valero et al., 2005). How physical environments modulate wine-related microbiota are affected by climate and weather, soil, and anthropogenic practices is considered in the following sections.

### Sampling and Methodologies

It is noteworthy that a lack of standardised sampling strategies and analytical framework impedes comparisons among studies, hindering the global insight of microbial ecology in wine production. For example, the depth of soil, the volume size of soil and plant materials (roots, bark, leaves, flowers, grapes), as well as must conditions (grapes crushed under aseptic conditions or collected directly from commercial wineries), vary among studies and generate different datasets (Supplementary Table S1) (reviewed in Morgan et al., 2017). Standardised sampling procedures are indispensable to obtain sound data for microbial biogeography studies. Metagenomic methodologies, covering from DNA extraction, target genes for sequencing (e.g., 16S V4 region, ITS region) to bioinformatics pipelines (e.g., QIIME), can generate technical variation among individual studies [reviewed in Morgan et al. (2017) and Stefanini and Cavalieri (2018)]. The example set by the Earth Microbiome Project^1^ to standardise sampling and analysis could be a good solution to reduce the technical variation and help understand the contribution of biological variation, weather, climate and other general trends.

## Climate Affects Wine Quality Via Microbiota

Climate, the long-term weather pattern of an area, is a profound element determining wine styles and regional characteristics and thus wine quality. Cooler climates are better suited to producing light and delicate wines, while warmer climates tend to shape heavy and rich flavour profiles. The climate impacts viticulture and wine quality through temperature, precipitation, and solar radiation (Van Leeuwen and Seguin, 2006). In viticulture, climatic influences are recognised at multiple geospatial scales. Macroclimate, or regional climate, is largely determined by latitude and altitude but also modified by moderating influences from water such as seas or large lakes (Figure 2; Van Leeuwen, 2010; White, 2015). The local climate of a particular vineyard is given as the mesoclimate, which is determined primarily by topography including altitude, aspect, and slope to impact upon wine quality and style (Figure 2; Gladstones, 1992; Van Leeuwen et al., 2004; Bramley et al., 2011). For example, vineyard orientation can affect the warmth and sunlight interception of grapevines, and steeper slopes benefit even more from this influence. At the smallest scale, microclimate, the temperature, humidity and solar variations within the canopy and between vines, may be affected in part by soil conditions and leaf shading and manipulated by canopy management (Figure 2; Smart and Robinson, 1991; Van Leeuwen and Seguin, 2006). Here, we highlight the microbial presence at these different scales to disentangle the intersection of microbes and climate and how this influences wine composition and style.

### Macroclimate

Microorganisms are mainly distributed by physical forces, such as air and wind (Zhu et al., 2017). Incorporation into clouds and precipitation and into nearby ecosystems increases their long-distance dispersal (Hamilton and Lenton, 1998; Figure 2). These forces shape microbial biogeographic patterns in a similar way to those of plants and animals (Hamilton and Lenton, 1998; Zhu et al., 2017). Climate, as the most important environmental factor of grapegrowing regions, exerts influences on local microbial incidence and persistence in both space and time (described as the vintage effect). At the macroclimate scale, grape must microbiota present regional distribution patterns which are significantly conditioned by local environments (e.g., temperatures and rainfall) and weakly affected by the vintage, for example maximum temperature and average low temperature are negatively associated with *Penicillium*, *Pseudomonas*, *Enterobacteriaceae*, and *Leuconostocaceae* (*O. oeni*) (Bokulich et al., 2014). Rainfall and humidity positively correlate with yeast *Hanseniaspora* and *Metschnikowia*, and negatively correlate with *Torulaspora, Saccharomyces* and *Meyerozyma* (Jara et al., 2016). As many of these species (in particular *Saccharomyces* and *O. oeni*) are the most abundant species present in a wine fermentation, is can be said that local climatic conditions can shape wine compositions by affecting their presence as seen in these association studies.

### Mesoclimate

At the mesoclimate level, or vineyard scale, consistency between vintages is one dimension of wine quality that is targeted for the *terroir* expression (Van Leeuwen et al., 2004; Beverland, 2006). Vintage variations substantially influence microbial communities in small geospatial scales (individual vineyards) rather than large scales (regions), with more stable fungal patterns observed between vintages (Bokulich et al., 2014). Vintage can significantly affect the biodiversity of yeast populations in the grape and must (Sabate et al., 2002; Vigentini et al., 2015). As a driver to shape mesoclimate, topographical features also exert influence on grape microbiota (Figure 2; Portillo et al., 2016). For example, *Oxalobacteraceae*, *Haemophilus*, *Sphingomonas*, and *Pseudomonas* were identified in grapes from east-facing vineyards, while *Streptococcus*, *Micrococcaceae*, *Staphylococcus*, *Enhydrobacter*, and *Aeromonadaceae* were shown as typical taxa of flat vineyards (Portillo et al., 2016).

### Microclimate

It could be argued that microclimate is the environment most likely to affect the presence, growth and activity of microbes. Modifying the grapevine leaf area through training, pruning, trellising, and defoliation alters canopy microclimate, in particular the solar radiation onto grapes and leaves, and to a lesser extent, by air movement in the leaf canopy (strongly affecting humidity) and temperature (Figure 2; Reynolds and Heuvel, 2009). The effect of light in the microclimate environment have been reported to affect a wide range of aspects of berry composition, and can strongly affect the colour and flavours present in the final wine (Haselgrove et al., 2000; Liu et al., 2015). However, studies on the effects of microclimate variability on microbial communities are rare. Fungal communities can be discriminated within vineyards and this effect was ascribed to microclimate variability, but no experimentally test has validated this correlation (Setati et al., 2012). From other studies it is clear that sunlight interception could affect the grapevine microbiome. For example, an aquatic yeast study suggested that *Cryptococcus* spp. and pigmented yeasts *Rhodotorula* spp. predominate in certain environments as they can produce photoprotective compounds (carotenoid pigments and mycosporines) to adapt to pelagic sites with high UV radiation (Brandão et al., 2011). These yeasts are also associated with grapevines (Sabate et al., 2002; Setati et al., 2012), and so a similar response is possible in the fruit zone affect the composition of fungal communities. Whether microclimate can influence the wine-related microbiome and influence resulting wine compositions remains speculative and deserves further study.

## The Influence of the Soil Borne Microbiome on Wine Composition and Quality

The soil substrate provides the grapevine with water and nutrients and soil type, composition and structure profoundly affects vine growth and development. Soil composition can affect the composition of wine, as wine can be related to its origin by tracking multiple major and trace elements from the soil to the wine (Almeida and Vasconcelos, 2003; Kment et al., 2005). Significant correlations were attributed to the movement of elements from the soil to grapes and resultant wines (Almeida and Vasconcelos, 2003). This contributes to our understanding of how soil geochemistry affects wine composition but has not been established mechanistically. An appreciation of the soil microbiota to vine health is relatively recent, and we review the knowledge in this area below.

### Soil Borne Microorganisms in Association With Grapevines

Grapevines live in biogeochemically diverse soils harbouring diverse microbiota that affect plant health and growth in beneficial, commensal, or pathogenic ways (Müller et al., 2016). Soil-borne microbes can affect crop yields and metabolite synthesis in other agricultural systems, and thus may shape wine colour, aroma, flavour, and quality. Serving as a reservoir for plant-associated bacteria (Zarraonaindia et al., 2015), soil borne bacteria can colonise plant organs by physical contact or travel from the rhizosphere toward the phyllosphere on the surface (epiphytes) or within plants (endophytes) (Figure 1; Chi et al., 2005; Compant et al., 2005; Compant et al., 2011; Martins et al., 2013; Ruiz-Pérez et al., 2016). For example, plant-growth-promoting (PGP) bacteria *Burkholderia* sp. strain PsJN can endophytically colonise Chardonnay plantlets and translocate onto the root surfaces, root internal tissues, and finally the internode and leaf (Compant et al., 2005). Some dominant taxa in the soil, such as *Gammaproteobacteria* (including *Pseudomonas* spp.) and *Firmicutes* (including *Bacillus* spp.) have been visualised by fluorescence *in situ* hybridisation as endophytes inside the flower ovules, and berries and seeds of Zweigelt grapevines (*Vitis vinifera*) (Compant et al., 2011).

As well as bacteria, the vineyard soil is one of the natural sources of fungi in musts (Morrison-Whittle and Goddard, 2018). Notably, identical genotypes of *S. cerevisiae* are found shared between soil and fruit niches within regions (Knight and Goddard, 2015). A vineyard field experiment suggested that *S. cerevisiae* yeasts can be adsorbed from the soil by roots and transported via vine to stems and surface of grapes, and finally entered fermentation musts (Mandl et al., 2015). This translocation process can enable soil borne microorganisms be a part of grapevine-associated microbiome that influence resultant wine quality and characteristics directly; these microbes can survive fermentation and release secondary metabolites affecting wine profiles or interact with fermentative yeasts or bacteria within the community. Of particular note for this discussion is the root associated rhizobia which has not been extensively studied in grapevines. This community is likely to have a profound affect as it was observed in rice plants (*Oryza sativa* L.) that an ascending endophytic migration of rhizobia starting on the rhizoplane surface, within root tissues, to reach the stem base, leaf sheath, and leaves, and this process benefits rice growth physiology afterward (Chi et al., 2005).

### Soil Properties and Interactions With Plants Shape Vineyard Microbiota

Biogeographic patterns have been observed in the microbial communities of vineyard soils (Table 1). Soil bacterial diversity and composition associate with the vineyard geographical location, with significant influences from soil physicochemical properties, such as soil texture, soil pH, temperature, moisture, carbon and nitrogen pools (Burns et al., 2015; Zarraonaindia et al., 2015). The majority of dominant taxa such as *Proteobacteria* (especially *Beta*- and *Gamma-proteobacteria*), *Bacteroidetes*, *Gemmatimonadetes*, and *Firmicutes* show higher relative abundance in soils with lower carbon or nitrogen contents, while abundance of *Actinobacteria* shows a negative trend (Burns et al., 2015). In addition, local climate (average annual precipitation) and topography (altitude, aspect, and slope) could indirectly influence soil microbial communities through their impacts on soil properties (Burns et al., 2015). Noticeably, fermentation-related bacteria in the musts are found in the vineyard soil as dominant taxa, such as *Firmicutes* (encompassing LAB) and spoilage bacteria *Acidobacteria* and *Proteobacteria* (Burns et al., 2015). As a reservoir of grapevine-associated bacteria, soil-borne microbes can shape phyllosphere bacterial assemblages, in particular grapes (Zarraonaindia et al., 2015). When the grapes are transferred into the winery and the microbiota persist in alcoholic fermentation, soil microbiota directly correlate with resulting wine metabolites.

Differentiation of vineyard soil bacterial community structure is reflected in the roots microbiome, with the relative abundance of several taxa occurring in a vineyard-depending manner. These taxa outcompete other bacteria for colonisation and/or are selected by grapevines. For example, PGP bacteria *Rhizobiales*, especially *Bradyrhizobium* spp., can benefit nitrogen fixation and antibiotic production that would promote plant growth, disease suppression and grape composition, thereby indirectly influencing wine quality and characteristics (Zarraonaindia et al., 2015). Within a vineyard, interactions between the root compartments (rhizosphere and root endosphere) and the rootstock can also exert a unique selective pressure enhancing niche differentiation of bacteria, in particular on the taxa with PGP potential, for example by producing indole acetic acid which affect plant hormone balances (Marasco et al., 2018).

Vineyard niches (soil, bark, fruit) account for approximately 40% of fungal populations in the musts and ferments. The clear similarity of fungi is between musts and grapes, that communities are dominated by fermenting yeasts (e.g., *S. cerevisiae* and *Hanseniaspora uvarum*) and filamentous fungi (*Aureobasidium pullulans*, *Cladosporium* spp.) (Morrison-Whittle and Goddard, 2018). While *Saccharomyces* spp. and wine spoilage species are mostly present in low numbers and in low frequencies in the soil, the most abundant genera (*Amniculicola*, *Doratomyces*, *Endocarpon*, and *Tricellulortus*) do not appear to be directly involved in wine production (Barata et al., 2012). In addition, regional delineations of fungal communities were observed in musts but not in the vineyard habitats at the regional scale (Morrison-Whittle and Goddard, 2018). Interestingly, significant differentiation of small-scale fungal communities was observed in a vineyard system (distance < 2 km), although with no vineyard demarcation between fermenting populations of *S. cerevisiae* (Knight et al., 2019). In-depth insight in fungal ecology in vineyards will inform how grapevines recruit fungal communities during the annual plant cycle and how this affects the communities present on the grape. For example, incidence of fermenting yeasts on grapes changes during ripening (Barata et al., 2012), and can thus contribute to wine metabolite profiles.

## Anthropogenic Practices Affect Quality Wine Production by Microbial Modulation

The natural environment of the vineyard determines grape production and composition. Based on the local conditions, thoughtful human management can optimise wine quality and style across vintages (White, 2015). Wine producers select vineyard sites with favourable environmental conditions (especially climate and soil) for quality wine production, as well as grapevine cultivars that adapt to the local environment (Van Leeuwen and Seguin, 2006). The grapegrower manages the vine to achieve balance between vine vigour and yield and may use different training, pruning, trellising and canopy methodologies, to harvest a healthy crop, with a desired fruit composition and to optimise wine quality (Jackson and Lombard, 1993; Reynolds and Heuvel, 2009). Pesticides (including fungicides against downy mildew, powdery mildew and grey rot) and fertiliser application are common interventions in the vineyard, which are now embedded within viticulture management practices of conventional, organic and biodynamic systems. Some evidence exists to differentiate the superiority of microbial diversity of organic or biodynamic wines with respect to specific management practice (Ross et al., 2009; Pagliarini et al., 2013), yet spontaneous fermentation and related winemaking techniques (for example cold soak, limited SO_2_ usage) are thought to encourage showing the indigenous microbial diversity, and thus enhance wine *terroir* expression (Capozzi et al., 2015). Recent studies focusing on the wine-related microbiome offer new information to this area and provide more information to allow expression of local individuality for viticultural production.

### Viticultural Practices

Specific human interventions including pesticides, fungicides, and herbicides usage can affect microbial diversity in specific habitats in vineyards (Čadež et al., 2010; Fierer et al., 2012a; Perazzolli et al., 2014; Pinto et al., 2014; Chou et al., 2018). Here we highlight the effect of viticulture practices on the vineyard microbiology, and whether these microbes and their effects can persist into wine production to ultimately influence wine quality.

Viticulture practices can modify the belowground microbiome. Soil borne bacterial communities are structured with respect to commercial/organic/biodynamic systems, as mediated by shifts in soil resource pools, particularly carbon and nitrogen (Burns et al., 2016). Compost addition in organic and biodynamic vineyards increases overall bacterial diversity and alter the community composition, and can the effect can be enhanced by tillage and cover crop management. Similarly, soil fungal diversity and community composition of biodynamic vineyards differ from those which are conventionally managed (Morrison-Whittle and Goddard, 2017; Table 1). Biodynamic management enhance fungal diversity in the grapevines niches (bark, fruits) but this effect did not persist into the harvested juice and fermentation (Morrison-Whittle and Goddard, 2017).

The phyllosphere microbiota can be shaped by agricultural management, in particular fungicide usage (Gilbert et al., 2014). Conventional vineyards are usually treated with several agricultural chemicals, while organic/biodynamic vineyards only recieve sulphur- and/or copper-based formulations. Several studies have shown a higher microbial diversity in grapes present in organic and biodynamic vineyards for both yeasts (*Saccharomyces* and non-*Saccharomyces*) and total fungi (yeasts and filamentous fungi) (Setati et al., 2012; Martins et al., 2014; Setati et al., 2015), and this could be because chemical treatments reduce microbial richness and diversity associated with grapevines and wine (Pinto et al., 2014; Escribano-Viana et al., 2018). This effect can maintain in spontaneous fermentations from organic/biodynamic musts where higher yeast species richness and diversity is observed compared to musts from conventionally managed vineyards, and this is particularly true for fermentative yeasts species such as *H. uvarum*, *H. vineae*, *H. guilliermondii*, *Streptomyces bacillaris*, *Lachancea thermotolerans*, and *S. cerevisiae* (Cordero-Bueso et al., 2011; Bagheri et al., 2015). Regarding grapevine-associated bacteria, responses to viticulture practices are weak compared to fungi, especially grape surface bacteria showing more resilience than that of leaves (Schmid et al., 2011; Miura et al., 2017). Biodynamic berries are found rich in *Bacillales* including genera of *Lysinibacillus*, *Bacillus*, and *Sporosarcin*, which are typical microbes in manure (Mezzasalma et al., 2017), but their influences on wine composition are not clear. Heavy use of sulphur and copper fungicides decrease the biodiversity of yeasts and fungi in resultant fermentations (Milanoviæ et al., 2013; Grangeteau et al., 2017). Particular fungi are associated with differently managed vineyards, where *Basidiomycota* (especially *Cryptococcus*) is mainly associated with organic vineyards, while fermentative yeasts *Saccharomyces*, *Metschnikowia*, and *Hanseniaspora* were mainly found in conventionally managed vineyards (Grangeteau et al., 2017; Table 1). The yeast-like fungus *Aureobasidium pullulans* dominate phyllosphere fungi in organic/biodynamic vineyards (Schmid et al., 2011; Pancher et al., 2012; Setati et al., 2012; Martins et al., 2014; Setati et al., 2015), but tends to be present only in the early stages of fermentation. Some debate exists, as Kecskeméti et al. (2016) claimed conventional/organic/biodynamic practices in the same vineyard do not significantly influence grape microbial diversity. Inconsistency is not surprising, as local vineyard conditions, specific management practices and sampling methods differ in the surveys published.

### Management of Microbes During Winemaking

The wine industry has developed a series of methods to promote wine fermentations, of which the most effective methods are the inoculation of cultured *S. cerevisiae* strains and use of sulphur dioxide (SO_2_). Commercial fermentations are widely used to reduce the risk of spoilage and unpredictable wine composition, and to ensure a stable wine flavour. However, inoculated fermentations reduce the potential of microbiota to contribute to regional characteristics. Spontaneous fermentations, comprising a diversity of yeast species and *S. cerevisiae* strains originating from vineyard and winery, enhance wine regional expression (Capozzi et al., 2015). The diversity of yeast species present can have profound impacts on the flavour of the resultant wine. For example, non-*Saccharomyces* yeasts can produce and secrete several enzymes (esterases, β-glucosidases, proteases), to synthesise volatile compounds and playing a role in varietal aroma (Romano et al., 2003). *H. uvarum* can positively interact with *S. cerevisiae* to enhance fermentation (Romano et al., 2003). Interactions within *S. cerevisiae* populations provide regional microbial signatures positively correlating with wine aroma profiles (Knight et al., 2015). Pre-fermentative cold soak, a technique widely used in red wine production to favour wine colour, taste and mouthfeel attributes, can influence yeast population dynamics depending the temperature. For example, a cold soak at 14 ± 1°C can increases total yeast populations and favour growth of *H. uvarum* and *Candida zemplinina*, whereas cold soak at 8 ± 1°C favours growth of *S. cerevisiae* (Maturano et al., 2015; Table 1). SO_2_ treatment favours the early implantation and domination of *S. cerevisiae* and alters wine microbial diversity and fermentation progression in a dose-dependent manner (Bokulich et al., 2015; Grangeteau et al., 2017; Table 1). A concentration of 25 mg/L SO_2_ is ideal to stabilise the microbial communities, which inhibits the growth of LAB and *Gluconobacter* but not other bacteria and fungi at early fermentation, thereby maximising microbial diversity to benefit wine regionality expression. Much less “manipulating the *terroir*,” production of distinctive and quality wine is achievable through microbial manipulation in the winery.

### How Will Climate Change Affect the Incidence and Activity of Microbes Involved in Winemaking?

Human activity has dramatically affected the global climate and the weather patterns that grapevines experience (Olesen et al., 2011). Wine production is particularly sensitive to climate change as there is an inherent link between climate and wine quality and style. Matching grape varieties to a unique combination of climate and soil enables production of distinctive wines worldwide (Van Leeuwen and Seguin, 2006), but this is likely to change as the temperature and water profiles of specific areas change. Trends in grapevine phenology associated with global warming are widely reported, and report earlier maturation with undesirable influences on grape and wine aroma and flavour (Palliotti et al., 2014). For example, wine grapes have been ripening earlier in Australia in recent decades, driven by warming and declines in soil moisture (Webb et al., 2012). A deficit of water will affect production of colourful and flavoursome wines rich in phenolic substances in a warmer and drier future (Bonada et al., 2015). In the long term, climate change will affect the geographical distribution of viticulture and will require changing varieties grown to adapt to warming or providing artificial shading to reduce temperatures (Jones et al., 2005; Webb et al., 2012), whereas in the short term, management techniques may be able to mitigate negative impacts and include managing soil water content (irrigation), crop yield (pruning regime), and vine response (rootstock selection, leaf removal) (Webb et al., 2012).

Microorganisms involved in winemaking are not discussed in the context of climate change. Increasing temperature and drought would strongly affect the ability to grow grapes and wine production, but in largely unknown ways. One area where these effects will be felt is in the soil and its microbial composition and activity. Studies outside of agriculture show that soil pH, moisture, temperature and nutrient availability are the main drivers of microbial community assembly (Fierer et al., 2012b) and that soil fungi and bacteria occupy specific niches and respond differently to precipitation and soil pH, and therefore would respond differently to climate change on their diversity, abundance, and function potential (Bahram et al., 2018). For example, fungal networks are more stable under drought conditions than bacteria in a grassland ecosystem (de Vries et al., 2018). Warming increases soil bacterial populations but decreases diversity and changes the composition (Sheik et al., 2011). Warming also increases soil respiration (Luo et al., 2001), which can in turn reduce the abundance of *Actinobateria* as sensitive to high carbon dioxide production (Sheik et al., 2011). Soil water saturation affects microbial composition by changing oxygen availability and thus microbial respiration (Carbone et al., 2011). Drought can also increase the resistance of soil bacteria (Bouskill et al., 2013), for example *Actinobateria*, which is tolerant of low moisture conditions (Fierer et al., 2012b). While these studies have been performed outside vineyards, it is likely that the same overall principals are at play. We suggest that superimposing warming and drying mechanisms will affect vineyard soil microbiota abundance, diversity and functions, and thus change their capacity to support plant growth. Further studies are needed, but it is clear that understanding wine microbial biogeography under the changing climate will help wine industry to adapt to climate change and enable quality wine production in the future.

## Conclusion and Future Perspectives

Fundamental questions about how wine quality and distinctiveness can be derived from local environments have been very difficult to answer. Here, we present microbial biogeography as core to understanding wine regionality. As the driver of wine fermentation, microorganisms inhabit and adapt to local geography, climate, soil and anthropogenic practices. The climate shapes microbial geographic diversification at multiple scales thereby affecting wine compositions. Soil-borne microbiota shapes the grapevine-associated microbiota and physiology and ultimately the flavour of resulting wines, but the mechanism by which this occurs remains to be elucidated. Human management practices modify wine-related microbiota to improve quality wine production. However, our knowledge of how geographically diverse microbiota shape wine chemical and organoleptic characteristics is limited. In-depth insights have emerged from studies of environment-plant-microbe interactions and will inform us about how grapevines recruit their microbiome to maximise both nutrition and microbial diversity under local conditions. The grapevine microbiota can then be sensibly exploited in wine production by introducing and/or managing specific microbes in combination with optimised wine metabolites to sculpt fermentation consortia for quality and distinctive wine production. Anthropogenic climate change will have profound consequences on wine-associated microbiota and thus affect wine quality and style. We believe that carefully designed empirical experiments to unpick microbial ecology and wine metabolome, surveys, and international and interdisciplinary collaborations will be indispensable to obtain a comprehensive understanding of climate change and the future of wine industry.

## Author Contributions

DL wrote the first draft of the review. KH revised and added to the draft. All authors approved the final version of the manuscript.

## Conflict of Interest

The authors declare that the research was conducted in the absence of any commercial or financial relationships that could be construed as a potential conflict of interest.

## Footnotes

1 http://www.earthmicrobiome.org/
